# *Pyrenophora teres*: Taxonomy, Morphology, Interaction With Barley, and Mode of Control

**DOI:** 10.3389/fpls.2021.614951

**Published:** 2021-04-06

**Authors:** Aurélie Backes, Gea Guerriero, Essaid Ait Barka, Cédric Jacquard

**Affiliations:** ^1^Unité de Recherche Résistance Induite et Bioprotection des Plantes, Université de Reims Champagne-Ardenne, Reims, France; ^2^Environmental Research and Innovation (ERIN) Department, Luxembourg Institute of Science and Technology (LIST), Hautcharage, Luxembourg

**Keywords:** barley, *Hordeum vulgare* L., *Pyrenophora teres*, net blotch, plant growth promoting rhizobacteria

## Abstract

Net blotch, induced by the ascomycete *Pyrenophora teres*, has become among the most important disease of barley (*Hordeum vulgare* L.). Easily recognizable by brown reticulated stripes on the sensitive barley leaves, net blotch reduces the yield by up to 40% and decreases seed quality. The life cycle, the mode of dispersion and the development of the pathogen, allow a quick contamination of the host. Crop residues, seeds, and wild grass species are the inoculum sources to spread the disease. The interaction between the barley plant and the fungus is complex and involves physiological changes with the emergence of symptoms on barley and genetic changes including the modulation of different genes involved in the defense pathways. The genes of net blotch resistance have been identified and their localizations are distributed on seven barley chromosomes. Considering the importance of this disease, several management approaches have been performed to control net blotch. One of them is the use of beneficial bacteria colonizing the rhizosphere, collectively referred to as Plant Growth Promoting Rhizobacteria. Several studies have reported the protective role of these bacteria and their metabolites against potential pathogens. Based on the available data, we expose a comprehensive review of *Pyrenophora teres* including its morphology, interaction with the host plant and means of control.

## Introduction

Worldwide, net blotch caused by *Pyrenophora teres* Drechsler [anamorph *Drechslera teres* (Sacc.) Shoem] is a major foliar disease of barley (*Hordeum vulgare* L.) causing economic losses by reducing the grain quantity and quality. According to Smedegard-Petersen (1971), net blotch exists in two different forms: the spot and net form of net blotch (SFNB and NFNB), caused by *P. teres* f. *maculata* (*Ptm*) and *P. teres* f. *teres* (*Ptt*) respectively (Smedegård-Petersen, 1976). These two forms have been identified as similar morphologically, however, different at the genetic and pathophysiological levels (Campbell et al., 1999; Liu et al., 2011; Akhavan et al., 2016). *Ptt* forms dark-brown and longitudinal necrotic lesions, which can turn chlorotic (Lightfoot and Able, 2010), while *Ptm* is responsible for dark brown circular or elliptical spots with chlorosis on the surrounding leaf tissues (Gupta and Loughman, 2001; Jayasena et al., 2004). The differentiation of these forms has been reported in Sweden (Jonsson et al., 1997), France (Arabi et al., 1992), Western Australia (Gupta and Loughman, 2001), South Africa (Louw et al., 1996), and Western Canada (Akhavan et al., 2016).

The distinction between these two forms is also due to differences in fungal growth and in symptoms’ development (Lightfoot and Able, 2010). Indeed, compared to *Ptt, Ptm* germinates slowly. Additionally, *Ptm* forms more intracellular vesicles compared to *Ptt* and is responsible of the leaf cell death within the fungal penetration area. The hyphal growth of *Ptt* is more extensive than *Ptm* before the formation of appressoria on the leaf surface. The time required for *Ptm* infection is also shorter than *Ptt* (Liu et al., 2011). Therefore, *Ptt* feeds and infects as a necrotroph during the infection period and grows only intercellularly. Contrary, *Ptm* initially develops haustorial-like intracellular vesicles, feeding similarly to a biotroph, and then switches quickly to a necrotrophic growth. Thus, *Ptt* behaves as a necrotroph, while *Ptm* acts as a hemibiotroph (Lightfoot and Able, 2010). A recent study has demonstrated that *Ptm* has significantly higher necrotrophic and saprotrophic growth rates than *Ptt* (Ronen et al., 2019). Several toxins are produced by both forms of *P. teres* (Bach et al., 1979; Nukina et al., 1980; Barrault et al., 1982; Friis et al., 1991; Weiergang et al., 2002), namely proteinaceous toxins and low molecular weight aspergillomarasmine-derived toxins contributing to the necrosis and chlorosis (Sarpeleh et al., 2007, 2008). *Ptt* produces greater quantities of toxins in the culture medium (Lightfoot and Able, 2010). In addition, the toxin composition and amount are different and remain to be established *in planta*.

*Pyrenophora teres* f. *teres* and *Pyrenophora teres* f. *maculata* are morphologically very similar, while the disease symptoms are different. Recent studies have shown that they are two phylogenetically distinct species, which are considered to be genetically autonomous populations (Akhavan et al., 2016). A period of evolutionary separation has been suggested thanks to a study of intergenic regions (Ellwood et al., 2012). Primers were developed on the basis of Internal Transcripted Spacer (ITS) regions and they allow to identify and distinguish the two forms of *P. teres* (Leisova et al., 2006; Mclean et al., 2009). In several parts of the world, the genetic diversity and population of *P. teres* were explored by using random amplified polymorphic DNA (RAPD; Peever and Milgroom, 1994; Campbell et al., 1999, 2002), amplified fragment length polymorphism (AFLP; Rau et al., 2003; Leisova et al., 2005; Serenius et al., 2007), and simple sequence repeat (SSR) analysis (Keiper et al., 2008; Bogacki et al., 2010; Leišová-Svobodová et al., 2014). In addition, both forms of *P. teres* have cycles of sexual reproduction occurring on overwintering crop residues followed by multiple cycles of asexual reproduction during the vegetative season (Piening, 1968; Duczek et al., 1999). Therefore, *Ptt* and *Ptm* have a mixed breeding and an outcrossing mating system. Because of these characteristics, *Ptt* and *Ptm* fall into the category of pathogens having a high capacity to adapt to resistance genes of the plant host as well as to fungicides. The recombination between *P. teres* isolates can lead to multiple resistances, for example, towards several triazoles (Jalli, 2011; Poudel et al., 2018). Studies have reported that the sexual reproduction between *Ptt* and *Ptm* is inducible under laboratory conditions (Campbell et al., 1999; Jalli, 2011). However, other studies have indicated that, under field conditions, hybridizations between *Ptt* and *Ptm* are unusual or even absent (Rau et al., 2003, 2007; Serenius et al., 2007; Poudel et al., 2018).

The first genome assembly of *Ptt* was obtained using the Illumina Solexa sequencing platform leading to a 41.95 Mbp of total assembly size (Ellwood et al., 2010). There have since been additional genomes sequenced and deposited in publicly available repositories including 11 *P. teres* f. *teres* genomes and five *P. teres* f. *maculata* genomes (Wyatt et al., 2018; Wyatt and Friesen, 2020). The genome assemblies of both forms of *P. teres* were constructed from long DNA reads, optical and genetic maps. These genomes are highly collinear and each one is composed of 12 chromosomes. The *Ptt* genome is larger and more repetitive than the *Ptm* genome (Syme et al., 2018).

In 1973, Shipton and collaborators published the first review on barley net blotch. This review compiled available information concerning the repartition and importance of the disease (Shipton et al., 1973). From 1973 to 2011, other reviews have been published describing the disease epidemiology and the host resistance toward *Ptm* (Mclean et al., 2009; Liu et al., 2011). In 2020, Clare and collaborators reported the brief consensus maps for all loci published for both barley and *P. teres* (Clare et al., 2020). To the best of our knowledge, no review has yet described the pathology of the fungus in relation with biological means to increase the resistance of the host. Beneficial bacteria are attracting attention in light of their potential use in agriculture (Babalola, 2010; Dutta and Podile, 2010; Vejan et al., 2016, 2019; Ferreira et al., 2019; Kumari et al., 2019; Prasad et al., 2019; Kumar et al., 2020). The present review provides an overview of the existing knowledge on the interaction between *P. teres* and barley and summarizes the current and ongoing research on *P. teres*. It also presents the morphological description, development of the fungus and the interaction with barley, and synthesizes the knowledge on current means used to manage net blotch.

## Taxonomy History and Focus on *Pyrenophora* Species

Established in 1809, the genus *Helminthosporium* became the repository for a large number of described taxa (Alcorn, 1988). *Helminthosporium* species attack the graminaceous plants in temperate regions (Sampson and Western, 1940). For instance, in 1943, *Helminthosporium oryzae* destroyed 90% of the rice in India, leading to a famine situation (Padmanabhan, 1973). This virulence was due to an increase of (i) the mean temperature, (ii) the rainfall, and (iii) the relative humidity. Nowadays, the major climate-change factors, including the increasing CO_2_, lead to an increase in humidity and therefore to optimal conditions for fungal development (Luck et al., 2011; Mikkelsen et al., 2015).

In 1923, Drechsler distinguished *Helminthosporium* species with cylindrical conidia growing over the whole cell surface and species with fusoid conidia germinating only at their ends (Drechsler, 1923). In 1929, Nisikado proposed two subgenera based on the description of Drechsler: *Cylindro-Helminthosporium* and *Eu-Helminthosporium* (Nisikado, 1929; Shoemaker, 1959; Alcorn, 1988). Later, Luttrell (1963) highlighted the differences between *Helminthosporium sensu stricto* and *Graminicolous* species in the development of conidia and conidiophores. Depending on the sexual states, *Graminicolous* species were separated into three genera, *Bipolaris, Drechslera*, and *Exserohilum* or *Cochliobolus, Pyrenophora* and *Setosphaeria*, respectively (Ibrahim et al., 1966; Ariyawansa, 2014). The genus *Bipolaris* contains *Eu-Helminthosporium* of Nisikado and *Helminthosporium* for lignicolous species (Shoemaker, 1959; Alcorn, 1988). Leonard and Suggs (1974) established the *Exserohilum* genus for species having strongly protuberant conidial hilum (Alcorn, 1988). In 1930, *Cylindro-Helminthosporium* became a genus under the name of *Pyrenophora* by Ito (1930).

*Pyrenophora* accounts for 135 species listed in Index Fungorum (2014), most of which are not pathogenic to humans (Ariyawansa, 2014). *Gramineae* and, more specifically, *Hordeum vulgare*, are considered as the main hosts for *Pyrenophora*. Depending on the barley’s symptoms, different species of *Pyrenophora* can be distinguished. Table 1 summarizes the main species of *Pyrenophora* involved in barley diseases.

**Table 1:** Summary of the different species of *Pyrenophora*, pathogen of barley.

Teleomorph stage	Anamorph stage	Host	Symptoms	Bibliographic references
*Pyrenophora graminea*	*Drechslera graminea*	barley	leaf stripe	(Tekauz, 1983; Porta-Puglia et al., 1986)
*Pyrenophora japonica*	*Drechslera tuberosa*	barley	leaf spot	(Campbell et al., 1999)
*Pyrenophora teres* f. *teres*	*Drechslera teres* f. *teres*	barley	net form of net blotch	(Brown et al., 1993; Jalli and Robinson, 2000; Afanasenko et al., 2007)
*Pyrenophora teres* f. *maculata*	*Drechslera teres* f. *maculata*	barley	spot form of net blotch	(Campbell et al., 1999; Jayasena et al., 2004; Mclean et al., 2009; Lartey et al., 2012)

**Figure 1:**
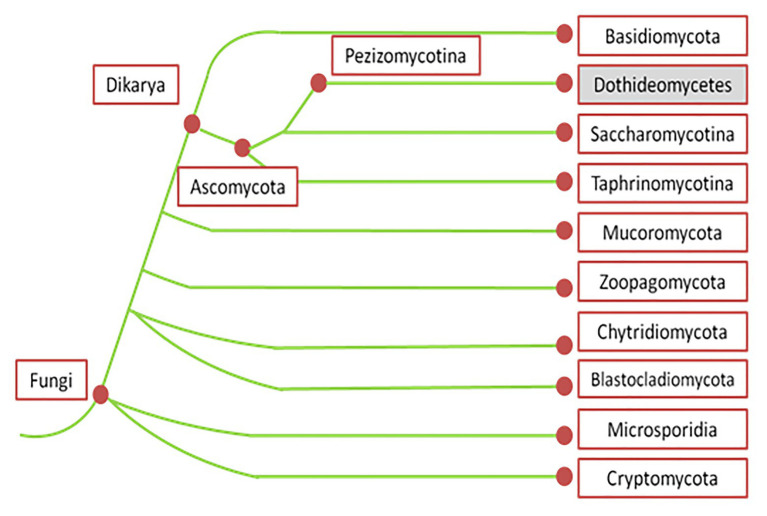
Phylogenetic tree of fungi according to MycoCosm portal (adapted from Grigoriev et al., 2014). According to this phylogenetic tree, *Pyrenophora teres* belongs to the kingdom *Fungi*, phylum *Ascomycota*, subphylum *Pezizomucotina*, and class *Dothideomycetes*.

*Pyrenophora graminea* [Ito & Kuribayashi; anamorph stage: *Drechslera graminea* (*Rabenhorst ex Schlechtendal* Shoemaker], is a seed-borne pathogen of barley causing leaf stripe (Porta-Puglia et al., 1986). *Pyrenophora japonica S.* Ito & Kurib. (anamorph stage: *Drechslera tuberosa* Shoemaker) is identified as the agent causing leaf spot symptoms on barley (Campbell et al., 1999). Net blotch caused by *P. teres* [anamorph stage: *D. teres (Sacc.) Shoemaker Drechsler*] belongs to the kingdom *Fungi*, phylum *Ascomycota*, subphylum *Pezizomycotina*, and class *Dothideomycetes* (Figure 1; Liu et al., 2011).

## Distribution and Economic Impact of Net Blotch

The ascomycete *P. teres* is the causal agent of net blotch on spring and winter barley. During the last decades, *P. teres* has spread throughout the world and ravaged crops in many countries: Australia (Gupta and Loughman, 2001; Mclean et al., 2010), Canada (Turkington et al., 2002; Akhavan et al., 2016), Europe (Dennis and Foister, 1942; Arabi et al., 1992; Plessl et al., 2005), South Africa (Smith and Rattray, 1930; Louw et al., 1996; Campbell et al., 1999), and the United States (Lartey et al., 2012). Net blotch causes important economic problems by reducing barley seed’s quality (Shipton, 1966; Plessl et al., 2005; Jayasena et al., 2007). For instance, in Australia, the economic losses are estimated to be $ 117 × 10^6^ per year (Murray and Brennan, 2010). In addition, yield losses might reach 40% in years with extensive rainfall in Germany (Plessl et al., 2005).

## Symptomatology

*Pyrenophora teres* causes disease and can infect leaves, stems, and kernels of barley (Liu et al., 2011). Like other plant diseases, symptoms’ appearance is dependent on the pathogen virulence, host genotype, and environment. Damages are different on resistant and susceptible varieties. Only few dot-like lesions are present with no development of a net-like pattern on highly resistant barley (Mclean et al., 2009; Liu et al., 2011).

**Figure 2:**
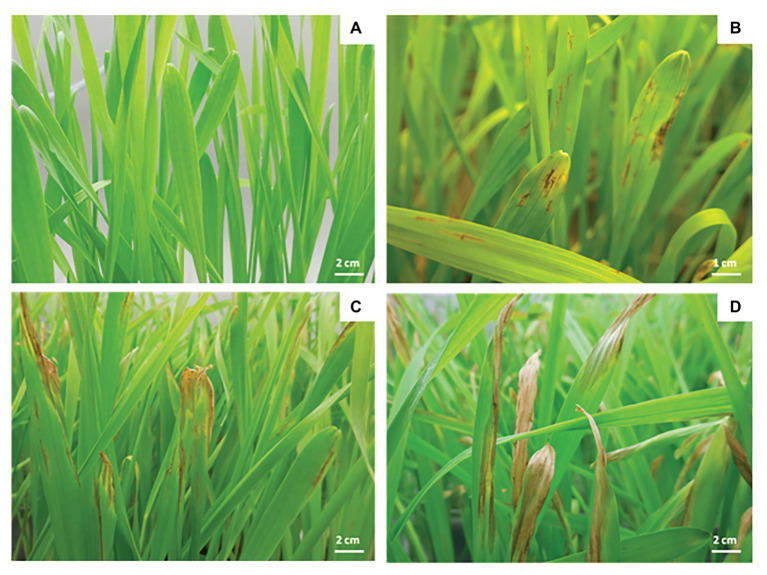
Symptoms caused by *P. teres* on Siberia barley leaves. Control **(A)**, 4 days after infection **(B)**, 7 days after infection **(C),** and 10 days after infection **(D)**.

On susceptible varieties, the disease evolves quickly as shown in Figure 2. Twenty-two hours post infection on a six row winter barley named Siberia, the first symptoms appear as brown necrotic spots on infected tissues increasing in size to form elliptical or fusiform lesions to 3 by 6 mm (Figure 2B; Keon and Hargreaves, 1983; Mclean et al., 2009). These necrotic lesions may be accompanied by chlorotic lesions of varying width (Figure 2C). Upon further fungal development, these chlorotic lesions might lead to the entire leaf’s death (Figure 2D). The first wilts appear 40 h after infection (Barrault et al., 1982; Arabi et al., 2003). Then, the oldest leaves start to wither, followed by the youngest.

These symptoms closely resemble those caused by *P. japonica* (Scott et al., 1991) and *Cochliobolus sativus* (Van den Berg, 1988), therefore examination of the conidia is often necessary to distinguish the pathogen agent (Mclean et al., 2009).

### Toxins’ Production

The symptoms caused by *P. teres* are partially induced by various toxins (Weiergang et al., 2002). The produced phytotoxic compounds include pyrenolides, pyrenolines, and three peptide alkaloids, aspergilomarasmine A and its derivatives (Muria-Gonzalez et al., 2020). Pyrenolines (A and B) and pyrenolides (A, B, C, and D) constitute general toxins affecting different plants (Nukina and Hirota, 1992; Sarpeleh et al., 2007) and induced only brown necrotic spots or lesions similar to those induced by the pathogen 72 h after inoculation (Sarpeleh et al., 2007). Pyrenolines A and B represent a class of bioactive metabolites produced by *P. teres* (Coval et al., 1990). While tested on both monocots and dicots, pyrenoline A shows no host specificity. In addition, pyrenoline B is also active on several plant species but at higher concentrations compared to pyrenoline A. Pyrenolides A, B, and C produced by *P. teres*, exhibit growth inhibiting and morphogenic activities towards other fungi. For instance, the application of pyrenolide C allows hyphal growth inhibition and the formation of many irregularly swollen hyphae in *Cochliobolous lunata* (Nukina et al., 1980).

**Figure 3:**
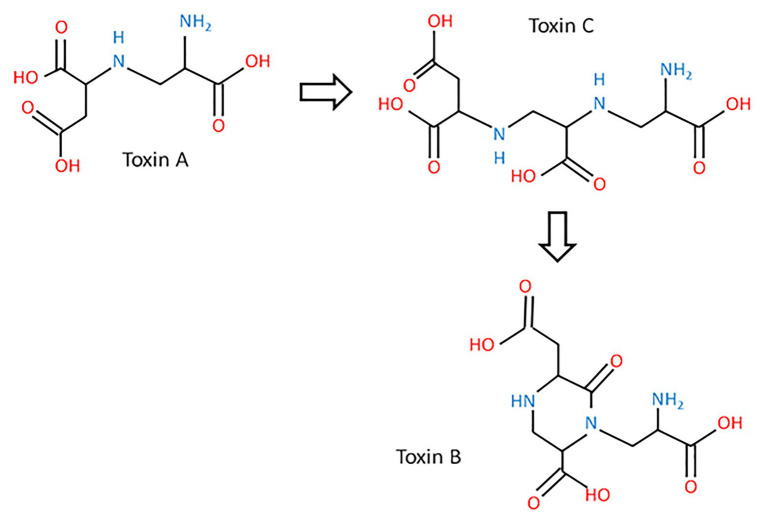
The biosynthetic pathway of aspergilomarasmine A (toxin C) and its derivatives (toxin A and toxin B) and their chemical structures (inspired from Friis et al., 1991).

In addition to pyrenolines and pyrenolides, *P. teres* also produces three other toxins, designated as N-(2-amino-2-carboxyethyl) aspartic acid, anhydrospergillomarasmine A, and aspergilomarasmine A, symbolized by A, B, and C, respectively (Barrault et al., 1982; Friis et al., 1991). These peptides are thermostable with a low molecular weight and cause chlorosis, rather than the classical net blotch necrosis, after infiltration of the pure compound (Muria-Gonzalez et al., 2020). The aspergilomarasmines were first described by Haenni et al. (1965), as metabolites of *Aspergillus oryzae* and *Aspergillus flavus* (Haenni et al., 1965). According to their chemical structures, toxin A might be a precursor of toxin C (Figure 3; Friis et al., 1991).

These toxins are involved in the development of the net blotch symptoms (Mikhailova et al., 2010). Toxin A is responsible for necrosis, while toxin B and C cause chlorosis (Weiergang et al., 2002; Mikhailova et al., 2010). Moreover, aspergilomarasmines A and B disturb the water balance of the plant cell. Their activities are enhanced by the presence of metal ions, especially ferric ions (Friis et al., 1991). Separated by electrophoresis, toxin C is the first detectable toxin, which is accumulated between 10 and 16 days after inoculation (Friis et al., 1991). The quantity of toxins produced by the fungus impacts the severity of symptoms (Sarpeleh et al., 2007; Mclean et al., 2009). Aspergilomarasmine A (toxin C) and its derivative (toxin A) are toxics at a concentration of 0.25 mmol/L, while the other derivative of aspergilomarasmine A, toxin B, is toxic at 1 mmol/L (Bach et al., 1979; Weiergang et al., 2002). These three peptides belong to host-specific toxins (HST; Stergiopoulos et al., 2013). Their activities are dependent on the age of plants with a higher level on young leaves (Sarpeleh et al., 2008). Toxins of *P. teres* are considered as virulence factors defined as the degree of damage caused to a host and not as pathogenicity factors representing the qualitative capacity of a pathogen to infect and cause disease on a host (Sacristán and García-Arenal, 2008; Jalli, 2011).

### Origin of Contamination

Characterized as a hemibiotroph, *P. teres* survives saprophytically between cropping seasons. The pathogen is present as mycelium on host crop residues, on seeds before sowing or wild grass species, forming a source of primary inoculum (Jordan and Allen, 1984; Brown et al., 1993). *Pyrenophora teres* is able to contaminate young shoots and the coleoptile (Jordan, 1981). The pathogenic factor and the quantity of primary inoculum from infected residues depend on several factors. Firstly, the environmental conditions and, more specifically, long periods of wet, increase the primary inoculum levels (Mclean et al., 2009). The amount of residues also directly impacts the disease intensity since the inoculum survives on infected residues. Secondly, the disease levels vary greatly depending on the cultural practice applied. Crop rotation, avoiding barley monoculture and eliminating or reducing primary inoculum in the field are means preventing the pathogen’s development (Liu et al., 2011). For instance, a minimum of 2 years between barley crops is required to prevent net blotch disease (Duczek et al., 1999). Concerning wild grass species, *P. teres* can infect *Agropyron, Bromus, Elymus, Hordelymus, Stipa*, and other *Hordeum* spp. (Mclean et al., 2009). These wild grass species can impact the epidemiology of net blotch, but they do not provide a significant additional inoculum to the next year’s crop (Brown et al., 1993).

### Life Cycle of *Pyrenophora teres*

**Figure 4:**
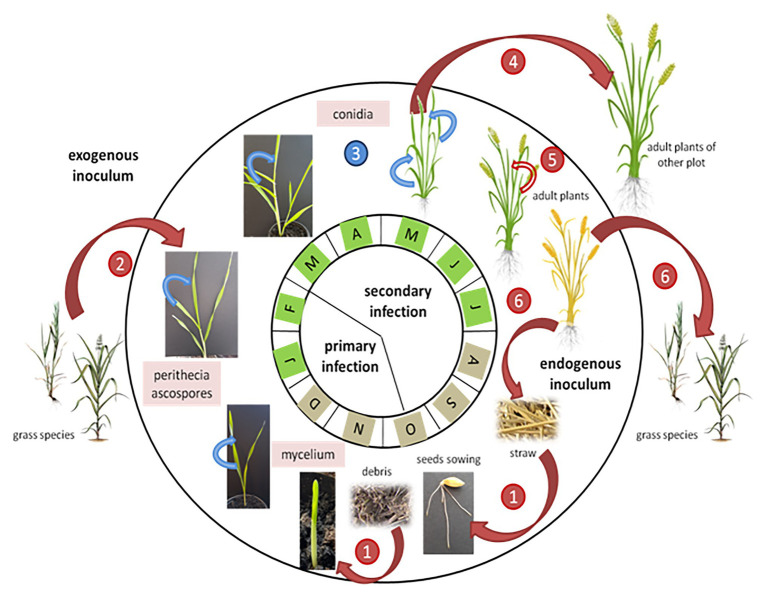
Dynamics of net blotch epidemics adapted from Suffert et al. (2011). Red arrows indicate *P. teres* wind-dispersed infections and blue arrows indicate splash-dispersed infections. The months with a brown color indicate that the source of inoculum comes from swarming debris on the soil, while the months with a green color indicate a source of inoculum mainly from the aerial parts of the plants. The numbers indicate the pathogen’s infection stages: 1: infection by *P. teres* ascospores present on infected barley debris; 2: mycelium present on grass species infects barley young plants; 3: the net blotch disease progresses from the bottom to the top of the barley plant; 4: disseminated by the wind, conidia contaminate other barley plants; 5: heavily infected crops show abortion of the ear; and 6: *Pyrenophora teres* colonizes the senescent tissues and produces perithecia on straw and grass species.

**Figure 5:**
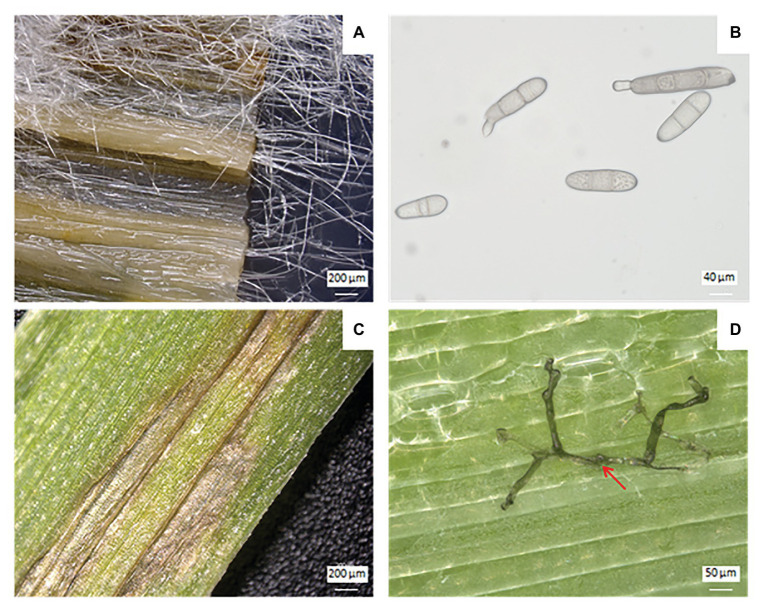
Vegetative and reproductive forms of *P. teres* and its symptoms on barley leaves. Mycelium of *P. teres* in barley leaf deposited on PDA medium **(A)**, conidia of *P. teres* **(B)**, chlorosis and necrosis symptoms on barley leaf caused by *P. teres* **(C)**, and penetration of mycelium (red arrow) through barley leaf **(D)**.

The *P. teres* lifecycle implies both an asexual and a sexual stage (Figure 4; Jalli, 2011). Conidia are produced during the asexual stage, whereas the sexual stage involves reproduction between isolates of compatible mating types and genetic recombination to produce ascospores (Fowler et al., 2017). The asexual stage occurs during the summer period on residues of the previous barley crop, and triggers the infection in autumn. At the end of the growing season, the fungus produces dark, globosely shaped pseudothecia, 1–2 mm in diameter (Figure 5A; Mclean et al., 2009). As sexual organs, pseudothecia represent the teleomorph or perfect state of *P. teres*. Once mature, pseudothecia produce asci containing three to eight ascospores measuring 18–28 μm × 43–61 μm, light brown and helical in shape (Liu et al., 2011). The ascospores production is temperature-dependent with an optimum between 15 and 20°C (Jordan, 1981; Mironenko et al., 2005). The spores have three or four transverse septa (Figure 5B). These ascospores are released on infected residues (Figure 5C) until spring constituting the primary inoculum (Duczek et al., 1999). These ascospores are dispersed into the air by the wind, or are splash-dispersed by the rain (Figure 4; Deadman and Cooke, 1989).

Following the primary infection, *P. teres* produces conidia measuring 30–174 μm × 15–23 μm constituting the asexual cycle (Liu et al., 2011). Generally, conidia are elongated with rounded ends. This secondary infection sets in 14–20 days after the primary infection and increases the severity of the disease (Mclean et al., 2009). The conidia, considered as the anamorph stage or imperfect stage of *P. teres* are found on conidiophores, solitary or grouped by two or three and swollen at the base (Mclean et al., 2009). Conidiophores need light to develop whereas conidia appear in the darkness (Wright and Sutton, 1990). Conidia of *P. teres* are recognizable from other pathogens due to their inside septum (Alcorn, 1988). The conidia are qualified as viable when they present more than two segments, otherwise they will not germinate and cannot penetrate plant tissues (Figure 5D). Between April and August, conidia are disseminated by the wind and/or rainfall to surrounding barley plants, or carried for long distances to reach new barley fields initiating, thus, the secondary infection cycle (Figure 4; Duczek et al., 1999). The infection process begins with the germination of ascospores or conidia on leaves. After penetrating the outer epidermal cell wall of barley, *P. teres* develops within a large intracellular vesicle called the primary vesicle (Liu et al., 2011). Then, vesicles are formed inside the sub-stomatal chamber leading to haustoria, the secondary hyphae (Keon and Hargreaves, 1983).

Thus, the pathogen contaminates the leaf, particularly the epidermal cells, within 48 h post infection (Jørgensen et al., 1998). The disease progresses from the bottom to the top of the plant (Figure 4). Further, the severity of damages is lower in older plants. Indeed, the older plants have a thicker cuticle limiting the penetration of the pathogen and have a greater capacity for producing antifungal substances (Khan and Boyd, 1969; Douiyssi et al., 1998). Once secondary infection is completed, *P. teres* colonizes the senescent tissues and produces pseudothecia on straw or weed residues (Figure 4; Liu et al., 2011).

## Methods for the Disease Assessment

Field trials can be tested, known as “hill summer,” to follow the development of the disease and to test resistance to net blotch in different barley cultivars. For hill summer trials, barley seeds are sown in hill plots at a distance between hills of 50 cm at the beginning of August. *Pyrenophora teres* inoculation is conducted by distributing naturally infested straw debris before sowing (early August; König et al., 2013; Vatter et al., 2017).

The evaluation of the disease in the laboratory and under controlled conditions is as follows: the barley plants are grown in controlled enclosure (temperature, humidity, and light) following a cycle of 23°C day/22°C night, 80% relative humidity, 14/10 h day/night photoperiod. For example, the *P. teres* pathogen can be sprayed with a sprayer on the leaves at different stages of growth after sowing in order to follow the disease development. For the control condition, the barley plants are sprayed with a sterile water solution. As soon as the pathogen is inoculated, the barley plants are placed in a hood to increase the humidity level, allowing the pathogen to improve its development. This same experiment can be carried out in a greenhouse under less controlled conditions, i.e., with a natural photoperiod.

**Figure 6:**
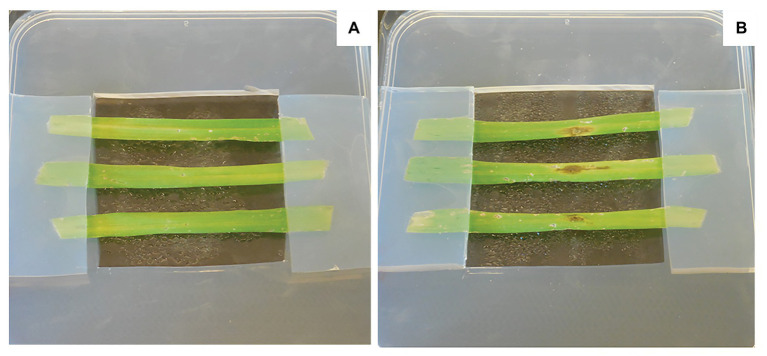
Assessment on detached leaves. The barley leaves were disinfected in an ethanol bath at 70°C followed by rinsing in three successive sterile water baths. All the leaves were cut to the same length then placed on agar-agar medium. These leaves were then wounded with a wooden pick. A volume of 10 μl was deposited at the level of the wound containing sterile water for a control condition **(A)** or *P. teres* spores at a concentration of 10^5^ spores.ml^−1^ for the infected condition **(B)**.

The disease can also be followed on detached leaves (Figure 6). Indeed, the detached plant leaf assay is a rapid technique of assessment under controlled conditions. For this experiment, barley leaves are cut to equal lengths and then placed on filter paper or on an agar-agar medium deposited or poured into a sterile plastic Petri dish. The barley leaves can be kept taut by means of agar–agar bands. Then, the inoculation is carried out by depositing 5–10 μl of a suspension of *P. teres* spores on previously injured barley leaves (Deadman and Cooke, 1986; El-Mor et al., 2017).

The infection response based on the measurement of individual lesion sizes (dimension; mm) for each second leaf was assessed 15 days after inoculation of *P. teres*. The 0–5 scale was used in this methods, where the scores 0–5 indicate resistant and increasingly susceptible barley phenotypes (Arabi and Jawhar, 2010). The disease severity for net blotch was scored on a dozen barley plants using modified Saari and Prescott’s double-digit scale (D1D2, 00–99) scoring method, which was based on the severity scale to assess foliar diseases in cereals. The first digit (D1) represents the relative height of the disease on the plant and corresponds to the vertical disease progression. The second digit (D2) refers to the severity measured as diseased leaf area (Saari and Prescott, 1975; Jalata et al., 2020).

## Genetics of the Interaction Between Barley and *Pyrenophora Teres*

**Figure 7:**
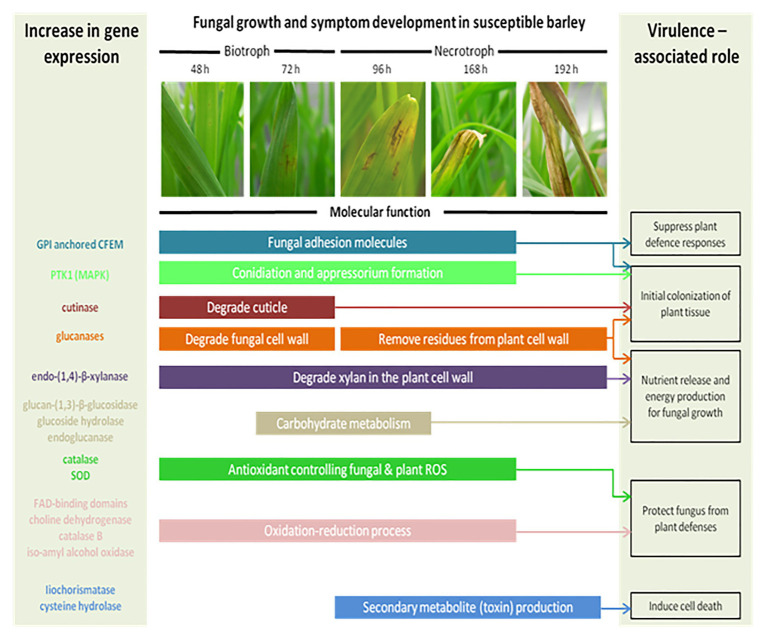
The schematic model of the fungal growth and symptoms’ development in a susceptible barley leaf. Each enzyme occurs at a specific time of infection. At 24 h, the spores germinate and form an appressorium through the plant epidermis supposing a biotrophic stage. By 96 h, *P. teres* is growing inside the barley leaf and develops chlorosis and necrosis suggesting a necrotroph stage. Colored bars represent the genes expressed during the plant/pathogen interaction and describe their molecular function(s) (Inspired from Ismail and Able, 2017).

At the molecular level, several mechanisms are involved during the interaction between *P. teres* and barley. In many fungal diseases, the infection cycle begins with the development of penetration structures triggered by the perception of chemical and/or physical signals from the plant surface. During the initial interaction, the mitogen-activated protein kinase (MAPK) signal transduction pathway plays crucial roles in the pathogenesis process. In *P. teres*, the MAPK *PTK1* gene allows the conidiation, appressorium formation, and pathogenicity on barley (Figure 7; Ruiz-Roldán et al., 2001). Other proteins containing a common domain in several fungal extracellular membrane proteins (CFEM), an eight cysteine-containing domain, are also required for appressorium development. CFEM-containing proteins could act as signal transducers or cell-surface receptors during host-pathogen interactions (Kulkarni et al., 2003; Ismail et al., 2014a).

*Pyrenophora teres* is able to modulate its cell wall in function of the vegetative or reproductive state and of the culture media used. Indeed, genes involved in the synthesis and remodeling of cell wall polysaccharides, namely chitin, β-(1,3)-glucan, mixed-linkage glucan, as well as endo/exoglucanases and a MAPK, varied in expression in *P. teres* spores and mycelium after cultivation on several media (Backes et al., 2020).

The cell wall of barley constitutes the first barrier to *P. teres*. To infect the plant, *P. teres* produces proteins degrading the plant cell wall (Muria-Gonzalez et al., 2020). These enzymes belong to cell wall degrading enzymes (CWDE; Figure 7). Each of these enzymes plays a role at several time points during the plant-pathogen interaction. *Pyrenophora teres* is characterized as a hemibiotrophic pathogen appearing as a biotroph during the first 24–48 h in contact with its host before switching to the necrotrophic stage (Ismail and Able, 2017). So, cutinase degrades the plant cuticle and participates to the initial colonization of plant tissues. Some of these proteins have been identified such as endo-(1,4)-β-xylanase and glucan-(1,3)-β-glucosidase precursors (Ismail et al., 2014b; Ismail and Able, 2016). Endo-(1,4)-β-xylanase is the key enzyme responsible for degradation of xylan, predominant hemicellulose in the plant cell (Beliën et al., 2006). Glucan-(1,3)-β-glucosidase precursor catalyzes the liberation of *α*-glucose from β-(1,3)-glucan, other principal component of plant cell wall (Boonvitthya et al., 2012). According to Ismail and Able (2017), genes coding for glycoside hydrolase family 43 protein, glycoside hydrolase family 105 protein, and endoglucanase-5 are highly expressed during the barley – *P. teres* interaction (Figure 7).

After the pathogen attack, the carbohydrate metabolism is impacted in plants. In barley, the sugar transporter genes are induced after pathogen attack (Williams et al., 2000; Bogacki et al., 2008). In the same way, the gene coding for a putative invertase allows to satisfy the increased metabolic demand on barley leaf tissue. An invertase-encoding gene, which cleaves sucrose to glucose and fructose, is induced in plants infected by fungal pathogens (Fotopoulos et al., 2003). The energy normally used for the primary metabolism is intended for defense mechanisms activated by the host plant against pathogens (Rojas et al., 2014). Consequently, these energy requirements for plant defense responses decrease the yield in barley (Shipton, 1966; Rojas et al., 2014).

Filamentous fungi may also produce effectors or proteinaceous toxins to facilitate the susceptible host colonization and notably cereal hosts (Gardiner et al., 2012; Ismail et al., 2014b; Muria-Gonzalez et al., 2020). Three proteins have been identified in *P. teres*: a cysteine hydrolase family protein, an endo-1,4- β-xylanase A, and an unknown secreted protein. The cysteine hydrolase family protein shows homology to an isochorismatase known as an enzyme suppressing plant defense (Ismail et al., 2014a).

During the interaction between *P. teres* and barley, a series of complex molecular and physiological processes activate and often lead to plant cell death, referred to as the hypersensitive reaction (HR; Goodman and Novacky, 1994; Greenberg, 1997; Heath, 2000; Lam et al., 2001; Greenberg and Yao, 2004; Mur et al., 2008; Künstler et al., 2016; Balint-Kurti, 2019). In the initiation of the HR, the oxidative burst triggers the production of reactive oxygen species (ROS). During the interaction between barley and pathogens, great quantities of ROS are produced resulting in an increase of antioxidant enzymes’ expression such as catalase and superoxide dismutase (*SOD*; Able, 2003). For instance, *HvCSD1*, a cytosolic *SOD* isolated from barley, is upregulated in barley tissues infected by *P. teres* (Lightfoot et al., 2017). The barley resistance to *P. teres* is correlated to this *SOD* activity increase and the *HvCSD1* seems to be important in the cytosolic redox status maintenance. Other genes involved in oxidation-reduction processes and defense mechanisms in plants including FAD-binding domains proteins, a choline dehydrogenase, or an iso-amyl alcohol oxidase were upregulated during the infection of barley by *P. teres* (Ismail and Able, 2017). Identified in 16 *P. teres* proteins, FAD-binding domain proteins are associated with multiple secondary metabolite pathways (Ismail and Able, 2016; Muria-Gonzalez et al., 2020). During infection of *Septoria nodorum* in wheat, the activity of catalase was also increased with the virulence of the pathogen and with the time post inoculation (Maksimov et al., 2013; Ismail and Able, 2017). Contributing to amine and polyamine biosynthesis, the choline dehydrogenase plays a role in barley infection by *P. teres*. The isoamyl alcohol oxidase produces aspergilomarasmine-derivatives, a toxin responsible for the chlorotic symptoms observed and described above.

## Net Blotch Disease Management

### Chemical Control

Causing 70% of cereal diseases, fungi are commonly controlled by fungicides. These products aim to ensure yield and to secure the quality of the harvest (Bartlett et al., 2002). Fungicides of the quinone outside inhibitors (QoI), the succinate dehydrogenase inhibitor (SDHI), and azole or demethylase inhibitor (DMI) classes are used as site-specific systemic fungicides (Mair et al., 2016). The foliar fungicide application effectiveness to control net blotch has been largely carried out (Sutton and Steele, 1983; Jayasena et al., 2002; Mclean et al., 2009). First studies have shown that triazole-based fungicides by pulverization allowed to control net blotch (Sutton and Steele, 1983; Van Den Berg and Rossnagel, 1990; Jayasena et al., 2002). Triazoles, known as DMI (propiconazole and prothioconazole), inhibit dimethylation between substrates that are necessary for the biosynthesis of ergosterol in fungi. In addition, SDHIs are also used to reduce the disease severity. In 1969, carboxin was the first SDHI fungicide launched, followed by several other SDHIs with a narrow spectrum of activity. The target of SDHI fungicide is the succinate dehydrogenase (SDH) enzyme or succinate ubiquinone oxidoreductases, which play an essential role in the tricarboxylic cycle and the mitochondrial electron transfer chain (Rehfus et al., 2016). The strobilurins, a new class of broad-spectrum fungicides, have been adopted these last years for net blotch control (Bartlett et al., 2002). Strobilurin fungicides were inspired by natural fungicidal derivatives of β-methoxyacrylic acid (Bartlett et al., 2002). Belonging to QoI (pyraclostrobin and picoxystrobin), strobilurins are natural substances isolated mainly from fungi and more specifically, *Basidiomycetes*. The strobilurin name is derived from the fungi genera *Strobilurus* (Balba, 2007). First introduced to the market in 1996, strobilurins inhibit mitochondrial respiration by blocking electron transfer at the level of cytochromes *b* and *c* (Bartlett et al., 2001; Gisi et al., 2002; Balba, 2007).

The antifungal efficacies depend also on the period of their application and how they are applied, as well as on the plant growth stage (Van Den Berg and Rossnagel, 1990). Seed treatments were successful if applied early in the season corresponding at Zadoks growth stage 23–24, but less at later growth stages (Martin, 1985). Barley seeds are considered as a source of inoculum for the ascomycete *P. teres*. The severity of the barley net blotch is reduced when a fungicide seed treatment is applied (Martin, 1985). Seed treatment effectiveness depends on fungal sensitivity, chemical fungitoxicity, and seed coverage quality. Iprodione is the fungicide providing the best control of dematiaceous fungi (*Bipolaris* and *Drechslera*) on seeds (Reis et al., 2012). Another study demonstrates the efficiency of one application of propiconazole at spike emergence for the management of net blotch (Sutton and Steele, 1983). A correct application of fungicides before the emergence of the flag leaf and the ear aims to protect the photosynthetic potential of the top four leaves, which contribute to 72% of the total yield (Mclean et al., 2009). A single application of propiconazole is not enough when the pathogen progresses quickly. A recent study demonstrates that two applications with the combination pyraclostrobin and epoxiconazole improved net blotch control and increased the yield in both experimental years (Stepanovic et al., 2016). Belonging to QoI, metyltetraprole is a new fungicide, which is effective against important cereal diseases, including net blotch (Suemoto et al., 2019). Further, the metyltetraprole suppresses succinate-cytochrome c reductase activity in QoI susceptible *P. teres*.

With time, resistant strains to these products have emerged (Jørgensen and Olsen, 2007). In Europe and in Australia, *P. teres* developed a resistance to DMI fungicides (Peever and Milgroom, 1993; Mair et al., 2016; Rehfus et al., 2016). Shortly after the first QoIs uses, resistant isolates to these antifungal products were detected in field populations (Gisi et al., 2002). More specifically, in 2003, resistance to QoI fungicides in *P. teres* was detected in France, Sweden, and Denmark. The resistance mechanism to QoIs has been identified as mutations in the mitochondrial target gene, cytochromes *b* (Sierotzki et al., 2007). In *P. teres*, this mutation has been described as a substitution of phenylalanine to leucine at amino acid position 129 (Sierotzki et al., 2007). To conclude, the fungicide exerts a selection pressure, which leads to the selection of isolates, which have a mutation providing fungicide resistance, while susceptible isolates will be eliminated. There is a subsequent increase in the number of resistant individuals in the population. Successive rounds of fungicide use repeat the selection of resistant isolates, which leads to the increase of the resistance mutation in the population each time the fungicide is used. Eventually, the resistant isolates will dominate the population and the effectiveness of the fungicide will be reduced (Gisi et al., 2000).

In addition to the resistance among several fungal species, azoles use has also been affected by a restriction with a wide range of significant toxicities, including hallucinations, hepatotoxicity, and QTc prolongation (Thompson et al., 2009; Gintjee et al., 2020). Faced with these problems, varietal selection, preventive agronomic measures, and biocontrol agents might be considered as alternative solutions to fungicides products.

### Host Plant Resistance

Cereals are selected according to their disease resistance (Jonsson et al., 1998). By definition, a resistant genotype is characterized by having the least and smallest visible foliar lesions, a fungal restricted growth on the infected leaf tissue and an increased production of antifungal products by barley leaves (Graner et al., 1996). Several studies have demonstrated the existence of resistance genes and loci to net blotch depending on the different forms of *P. teres*.

Geschele (1928) has first demonstrated the resistance to *P. teres* f. *teres* to be quantitatively inherited (Clare et al., 2020). The genetic control of resistance to *P. teres* in barley was first conducted in United States in 1955 (Afanasenko et al., 2007). In 1955, the first gene *Pt1* conferring the barley resistance to *P. teres* was found by Schaller. Later, two additional loci, designated *Pt2* and *Pt3* were identified by Mode and Schaller in 1958 (Mode and Schaller, 1958; Graner et al., 1996). Using different molecular techniques, several studies have identified net blotch resistance genes or quantitative trait loci (QTL) on all seven barley chromosomes (Mode and Schaller, 1958; Steffenson et al., 1996; Manninen et al., 2000; Gupta et al., 2011; Clare et al., 2020). Major QTL have been identified on barley chromosomes 1H (Bockelman et al., 1977; Manninen et al., 2000, 2006), 2H (Bockelman et al., 1977; Williams et al., 1999; Ma et al., 2004; Adawy et al., 2013; Tamang et al., 2019), 3H (Bockelman et al., 1977; Graner et al., 1996), 4H (Friesen et al., 2006; Grewal et al., 2008; Adawy et al., 2013; Islamovic et al., 2017), 5H (Manninen et al., 2006; Adawy et al., 2013), 6H (Ma et al., 2004; Friesen et al., 2006; Abu Qamar et al., 2008; Grewal et al., 2008; Gupta et al., 2011; Adawy et al., 2013), and 7H (Williams et al., 1999; Grewal et al., 2008; Mclean et al., 2009; Tamang et al., 2019). Localized on chromosome 6H, the *Rpt5* locus has been reported by several studies and is considered to be essential in the *P. teres* f. *teres* – barley interaction (Clare et al., 2020). According to several studies, the majority of the markers significantly associated with NFNB resistance localize to the centromeric region of chromosome 6H (Abu Qamar et al., 2008; Adawy et al., 2013; Richards et al., 2016). In the same way, the high-resolution mapping of a dominant susceptibility locus located in the centromeric region of barley chromosome 6H has been described using markers (Richards et al., 2016). Therefore, these results indicate the importance of this region. In addition, the *Rpt7* locus confers resistance to *P. teres* f. *teres* in barley on the chromosome 4H. Recently, 449 barley accessions were phenotyped for *P. teres* f. *teres* resistance in greenhouse trials. Using genome-wide association, the results identified 254 marker-trait associations corresponding to 15 QTLs. Four of these regions were new QTL not described in previous studies and are located on chromosome 3H at 233–350 Mpb, 5H at 579 Mbp, 6H at 406–410 Mpb and 7H at 5 Mbp, respectively (Novakazi et al., 2019).

Initially, the genetics conferring resistance to *P. teres* f. *maculata* contained three major designated loci and therefore has been considered less complex to compare the *P. teres* f. *teres* – barley interaction (Clare et al., 2020). Designated as *Rpt4, Rpt6*, and *Rpt8*, these three major loci confer in barley a resistance to *P. teres* f. *maculata*. The *Rpt4, Rpt6*, and *Rpt8* loci are localized on chromosome 7H, 5H, and 4H, respectively. Burlakoti et al. (2017) revealed the effect of two- and six-row barley, and concluded that the two-row barley (13%) resistant to *P. teres* f. *maculata* was less than the six-row barley (43%) tested.

The use of resistant varieties is proving to be one of the most effective methods with the least environmental impact. Nevertheless, this selection has a high financial cost for farmers (Mclean et al., 2009).

### Preventive Agronomic Measures

Based on the life cycle described earlier in this review, three sources can form the primary inoculum of which infected seeds, crop debris, and straw residue. Therefore, the first step to control net blotch is the deletion of the primary inoculum of *P. teres* by sowing healthy seeds (Jalli, 2011). After harvesting barley kernels, debris and straw residues are other sources of primary inoculum. A study has demonstrated that amounts of residue infested can increase disease intensity and thus reduce the yield (Adee, 1989). To limit the primary inoculum and reduce straw debris, straw may be baled and removed from fields leaving standing stubble after the harvest (Jordan and Allen, 1984). In addition, some farmers practiced open field burning a few years ago. However, due to the pollution (smoke and smuts) generated the loss of organic matter and damage to wildlife and hedges, this practice of burning is now prohibited. Today, new means exist to eliminate the quantity of inoculum present on the straw residues such as chopping and burying (Jordan and Allen, 1984).

In addition to these measures to limit the sources of inoculum, preventive agronomic measures play an essential role in the management of net blotch. Crop rotation is beneficial to reduce the severity of the pathogen. A minimum of 2 years between barley crops is required to prevent net blotch (Duczek et al., 1999). The type of seedling has also an influence on the severity of net blotch. Today, agricultural trends aim to reduce field trips with direct seeding for example. Consisting of sowing the seeds directly in undisturbed soil, direct sowing increases the quantity of *P. teres* present on crop debris and straw residues (Jordan and Allen, 1984; Mclean et al., 2009). Therefore, direct sowing reduces the cost of production but increases *P. teres* severity (Jørgensen and Olsen, 2007).

### Biological Control

The development of environmentally friendly methods based on biological agents is of great interest in the context of the establishment of a sustainable agriculture (Cazorla et al., 2007). The seeking and the selection of biological agents targeting the pathogen, plays an essential role in the success of biological control strategies (Cazorla et al., 2007). Biological control brings together a set of biocontrol agents including microorganisms (bacteria, fungus, and viruses), macroorganisms (birds, insects, and nematodes), as well as molecules derived from these organisms (natural substances and chemical mediators; Pérez-García et al., 2011). Biological control results from a combination of mechanisms, including competition for nutrients and space, production of antibiotics, and induced systemic resistance to pathogens.

To effectively limit pathogens, biocontrol agents need to rely on plant colonization strategies and maintain a high population density (Ghorbanpour et al., 2018). Competition for nutrients and the space between biological control agents and pathogens is considered the primary mode of action (Sharma et al., 2009). The antibiotics production and antibiosis phenomenon constitute the second mechanism, which controls the development and spread of pathogens (Sharma et al., 2009). The last means for biological control agents to limit the development of pathogens is by inducing systemic resistance. Following recognition with a beneficial organism, the plant can respond systemically and rapidly to the perception of the pathogen. The phenomenon called “priming” allows the plant host to set up more quickly and massively the various defense mechanisms against pathogens (Conrath et al., 2002).

In this review, we only highlight the bacteria defined as plant growth promoting rhizobacteria (PGPR) and used in biological control thanks to their beneficial effects.

The term “PGPR” was first coined in 1978 and these bacteria are classified into two groups. The first group includes bacterial strains able to colonize roots, enhance emergence, stimulate growth either directly, by the capability of synthesizing plant growth-promoting substance, or indirectly, by changing the microbial composition in the rhizosphere in favor of the beneficial micro-organisms (Ait Barka et al., 2000, 2002; Hardoim et al., 2008; Turan et al., 2012). They may induce a systemic resistance to pathogens and modulate the plant regulatory mechanisms through the production of hormones such as auxin, cytokinins, and gibberellins (phytostimulators; Van Loon et al., 1998; Ait Barka et al., 2000, 2006; Bloemberg and Lugtenberg, 2001). In addition, some beneficial bacteria fix atmospheric nitrogen, solubilize inorganic nutrients limiting plant growth, stimulate nutrient delivery and uptake by plant roots and improve nutrient and water management (biofertilizers; Babalola, 2010; Afzal et al., 2019). A second group prevents or decreases the deleterious effects of pathogens (Turan et al., 2012). These bacteria are used in agricultural practices against diseases in light of the improved plant performance under environmental stress and, consequently, of yield enhancement (Ait Barka et al., 2000). The biocontrol agents that are best-characterized belong to the genus *Pseudomonas, Streptomyces Paraburkholderia*, and *Bacillus* (Bloemberg and Lugtenberg, 2001; Beneduzi et al., 2012; Esmaeel et al., 2016, 2018; Shafi et al., 2017; Newitt et al., 2019). For instance, a study shows that *Bacillus substilis* has antagonist activity against the causal agent of *Fusarium* head blight in wheat under *in vitro* and greenhouse assays (Palazzini et al., 2016).

In the rhizosphere, a community of several strains is often more stable, suppresses a broader range of pathogens and sets up different mechanisms of biological control (Jetiyanon and Kloepper, 2002). The great potential for using bacteria has been shown as an alternative to fungicides in the management of plant diseases (Ait Barka et al., 2002).

The microorganisms are able to produce a wide range of antimicrobial peptides including small bacteriocins and fungal defensins (Waghu and Idicula-Thomas, 2020). In addition, these microorganisms are also able to produce secondary metabolites such as peptaibols, cyclopeptides, and pseudopeptides by non-ribosomal synthesis (Montesinos et al., 2012). The beneficial bacteria also produce antifungal antibiotics, named cyclic lipopeptides, which allow acting as antagonistic agents against pathogens. These molecules with low-molecular-weight are deleterious to the growth of other organisms (Beneduzi et al., 2012). Synthesized in a non-ribosomal manner, lipopeptides exhibit surfactant and antimicrobial activities due to amphiphilic features that have drawn attention (Cazorla et al., 2007). These antibiotic compounds are mostly produced by *Bacillus* species and *Burkholderia* and more specifically, *Paraburkholderia phytofirmans* species (Ongena et al., 2007; Pérez-García et al., 2011; Esmaeel et al., 2016, 2018).

### Use of Biocontrol Toward *Pyrenophora teres*

Concerning *P. teres*, many species of *Trichoderma* produce secondary metabolites like volatile organic compounds (VOCs) in the presence of *P. teres*. These VOCs have been identified as sesquiterpenes, diterpenes, terpenoids, and eight-carbon compounds. In addition, VOCs inhibit mycelium growth and lead to unpigmented mycelium with vacuolization (Moya et al., 2018). In the same way, *Trichoderma* isolates significantly decrease the severity of *P. teres*, up to 55% on barley seedlings, 70% on leaves and 77% on stems (Moya et al., 2020). Finally, the fungus *Clonostachys rosea* isolate IK726 reduces the infection caused by *P. teres* under controlled conditions (Jensen et al., 2016).

The use of biological agents is considered as one of the most promising methods for more rational and safer crop-management practices (Ongena and Jacques, 2008) since they reduce the inputs use and increase the plant vigor. Future experiments will confirm the biological control use to limit net blotch.

## Conclusion and Future Perspectives

This review provides a reference point on net blotch of barley by highlighting *P. teres* severity, the changing complexity during the interaction between barley and *P. teres* and the management of net blotch control. Net blotch has become a major foliar disease of barley in many countries of the world. Caused by the ascomycete *P. teres*, this disease causes significant grain yield loss and reduces grain quality. Net blotch develops quickly when the environmental conditions are optimal including long periods of wet and cultural practice used. Additionally, *P. teres* produces a large quantity of toxins improving its virulence factor. The variability of the fungal pathogenicity leads to the conclusion that symptoms’ occurrence is dependent on the host genotype, the pathogen virulence, and the environment. The net blotch control provides a significant challenge now and in the future. Chemical control, host plant resistance, and preventive agronomic measures are used for net blotch management. Among them, as an environmental-friendly means, the biocontrol agents appear as a promising tool towards a sustainable agriculture.

## Author Contributions

AB wrote the paper. All authors have proofread, amended, and approved the final version of the manuscript.

### Conflict of Interest

The authors declare that the research was conducted in the absence of any commercial or financial relationships that could be construed as a potential conflict of interest.
